# T-cell priming transcriptomic markers: implications of immunome heterogeneity for precision immunotherapy

**DOI:** 10.1038/s41525-023-00359-8

**Published:** 2023-08-08

**Authors:** Hirotaka Miyashita, Razelle Kurzrock, Nicholas J. Bevins, Kartheeswaran Thangathurai, Suzanna Lee, Sarabjot Pabla, Mary Nesline, Sean T. Glenn, Jeffrey M. Conroy, Paul DePietro, Eitan Rubin, Jason K. Sicklick, Shumei Kato

**Affiliations:** 1 Department of Hematology and Oncology, Dartmouth Cancer Center, Lebanon, NH USA; 2 Worldwide Innovative Network (WIN) for Personalized Cancer Therapy, Paris, France; 3 Division of Hematology and Oncology, Medical College of Wisconsin, Milwaukee, WI USA; 4 Department of Pathology, University of California San Diego, La Jolla, CA USA; 5 The Shraga Segal Department for Microbiology, Immunology and Genetics, Ben-Gurion University of the Negev, Beer Sheva, Israel; 6 Department of Physical Science, University of Vavuniya, Vavuniya, Sri Lanka; 7 Center for Personalized Cancer Therapy and Division of Hematology and Oncology, Department of Medicine, UC, San Diego Moores Cancer Center, La Jolla, CA USA; 8 OmniSeq Inc, Buffalo, NY USA; 9 Roswell Park Comprehensive Cancer Center, Center for Personalized Medicine, Buffalo, NY USA; 10 Division of Surgical Oncology, Department of Surgery, and Center for Personalized Cancer Therapy, University of California, San Diego, La Jolla, CA USA

**Keywords:** Tumour immunology, Genome informatics

## Abstract

Immune checkpoint blockade is effective for only a subset of cancers. Targeting T-cell priming markers (TPMs) may enhance activity, but proper application of these agents in the clinic is challenging due to immune complexity and heterogeneity. We interrogated transcriptomics of 15 TPMs (CD137, CD27, CD28, CD80, CD86, CD40, CD40LG, GITR, ICOS, ICOSLG, OX40, OX40LG, GZMB, IFNG, and TBX21) in a pan-cancer cohort (*N* = 514 patients, 30 types of cancer). TPM expression was analyzed for correlation with histological type, microsatellite instability high (MSI-H), tumor mutational burden (TMB), and programmed death-ligand 1 (PD-L1) expression. Among 514 patients, the most common histological types were colorectal (27%), pancreatic (11%), and breast cancer (10%). No statistically significant association between histological type and TPM expression was seen. In contrast, expression of GZMB (granzyme B, a serine protease stored in activated T and NK cells that induces cancer cell apoptosis) and IFNG (activates cytotoxic T cells) were significantly higher in tumors with MSI-H, TMB ≥ 10 mutations/mb and PD-L1 ≥ 1%. PD-L1 ≥ 1% was also associated with significantly higher CD137, GITR, and ICOS expression. Patients’ tumors were classified into “Hot”, “Mixed”, or “Cold” clusters based on TPM expression using hierarchical clustering. The cold cluster showed a significantly lower proportion of tumors with PD-L1 ≥ 1%. Overall, 502 patients (98%) had individually distinct patterns of TPM expression. Diverse expression patterns of TPMs independent of histological type but correlating with other immunotherapy biomarkers (PD-L1 ≥ 1%, MSI-H and TMB ≥ 10 mutations/mb) were observed. Individualized selection of patients based on TPM immunomic profiles may potentially help with immunotherapy optimization.

## Introduction

Immune interventions by cytokines, vaccines, checkpoint blockade, CAR T cells and other agents have been studied as approaches to treat cancer. These endeavors have led to multiple approvals of immunotherapeutics by the Food and Drug Administration (FDA), starting with immune-stimulatory agents such as interferons and interleukins^1,2^. More recently, a better understanding of the molecular mechanisms of suppressed immune response in the tumor microenvironment has enabled the discovery of more potent immunotherapy agents. In 2011, FDA approved the first immune checkpoint blockade (ICB)—ipilimumab—a monoclonal antibody targeting cytotoxic T-lymphocyte-associated protein 4 (CTLA-4), which gained authorization for advanced melanoma^3^. Subsequently, pembrolizumab, a programmed death 1 (PD-1) inhibitor, was approved as a treatment for advanced melanoma by FDA in 2014^4^, followed by approval of atezolizumab, a programmed death-ligand 1 (PD-L1) inhibitor, for advanced urothelial carcinoma in 2016^5^. As of 2021, one CTLA-4 inhibitor, four PD-1 inhibitors, and three PD-L1 inhibitors have been approved. These potent immunotherapies have impacted the treatment strategies of a large number of malignancies^6–8^.

Despite the success of ICB in the management of advanced cancers, only a portion of patients will respond. For instance, a result from an early phase trial of pembrolizumab for advanced non-small cell lung cancer showed an objective response rate of 19.4%^9^. To overcome this challenge, several response markers and comprehensive immune signatures have been exploited to select patients who would benefit from ICB, including PD-L1 expression status^10–13^, microsatellite instability (MSI)^14^ and tumor mutational burden (TMB)^15–17^. More recently, it was shown that other genomic abnormalities, such as *ARID1A* mutations and specific genomic signatures, are associated with favorable outcomes after ICB^18–21^.

Even with the best available response markers, the response rate is still only about 40–50% in biomarker-selected populations^22^. To improve the response rate, combination regimens such as ICB with chemotherapy^23^, targeted therapy^24^, or another type of ICB^25^ have been investigated. Another potential approach to improving immunotherapy outcomes involves performing clinical trials that are centered on stimulating cancer immunity through T cells. T cell priming refers to the process of the T cell activation following a primary recognition of specific peptide–major histocompatibility complexes (MHC), and leading to expansion of clones of differentiated effector cells^26,27^.

The genes associated with T cell priming are called T-cell priming markers; they include, but are not limited to, CD137, CD27, CD28, CD40, CD40LG, CD80, CD86, GITR, GZMB, ICOS, ICOSLG, IFNG, OX40, OX40LG, and TBX21 (Supplementary Table 1). The preliminary results from clinical trials of T cell priming to date are, for the most part, not very promising despite the strong scientific rationale; the response rate is ~0–20% in most of the trials with immune-stimulating factors. (Supplementary Table 2) One possible explanation for the limited response rate is the heterogeneity of cancer immunity. For instance, PD-L1 expression may vary widely in solid tumors, ranging from 0 to 100% even among the same histological type of cancer^11^. Moreover, immunogram complexity and heterogeneity, reflected by complicated and distinct RNA expression patterns of cancer immunity markers has been reported for other checkpoints as well^28^. Although some recent clinical trials have been designed based on each patient’s molecular features^29^, most of the clinical trials with immune-stimulating factors do not select the patients based on their immunogram.

We hypothesized that T-cell priming markers may exhibit heterogeneity between and within histologies.

Therefore, this study aimed to interrogate the transcriptomic diversity of T-cell priming markers across and within advanced cancer types, and to determine correlations with canonical immunotherapy markers such as PD-L1 expression, TMB and/or MSI status.

## Results

### Patient demographics

**Fig. 1:**
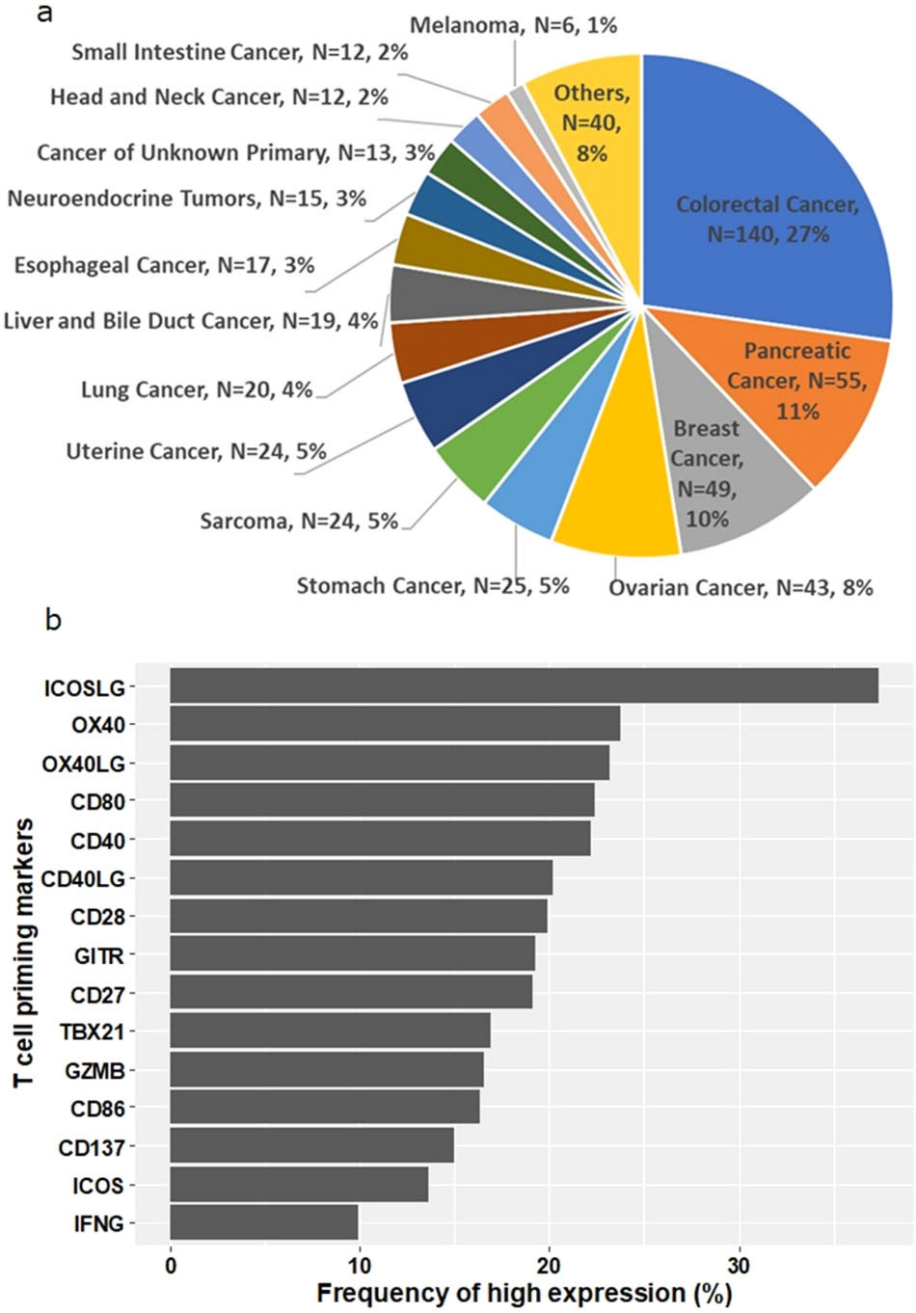
Baseline characteristics of the cohort (*N* = 514). **a** Pie chart of cancer types in the cohort (*N* = 514). Others include: cervical cancer (*N* = 5), bladder cancer (*N* = 4), gallbladder and extrahepatic bile duct cancers (*N* = 4), prostate cancer (*N* = 4), brain and nervous system cancers (*N* = 3), kidney and renal pelvis cancers (*N* = 3), squamous cell carcinoma of the skin (*N* = 3), thyroid cancer (*N* = 3), adrenal gland cancer (*N* = 3), lipomatous neoplasm (*N* = 2), mesothelioma (*N* = 2), basal cell carcinoma of the skin (*N* = 1), ocular melanoma (*N* = 1), primary peritoneal carcinoma (*N* = 1), and thymic cancer (*N* = 1). **b** Frequency of patients with high expression of T cell priming markers (*N* = 514). Horizontal axis represents the percentage of patients with high expression of each T cell priming marker. Transcript abundance was normalized to internal housekeeping gene profiles and ranked (0–100) to standardized values by comparing to a reference population of 735 tumors spanning 35 histologies. The expression profiles were stratified by rank values into “Low” (0–24), “Intermediate” (25–74), and “High” (75–100). See *Methods* as well.

We analyzed 514 samples from patients with a wide variety of advanced/metastatic cancers as summarized in Fig. 1a and Supplementary Table 3. The most common type of cancer was colorectal cancer (27.2%) followed by pancreatic (10.7%), breast (9.5%), ovarian (8.4%) and stomach cancer (4.9%). The median (range) of the patients’ ages was 60.8 years (23.9–93.3 years old). Sixty percent (*N* = 310) of the patients were women.

### Overview of RNA expression of T-cell priming markers among diverse cancers

Per the *Methods*, transcript abundance of the 15 T-cell priming markers was normalized to internal housekeeping gene profiles and ranked (0–100 percentile) to standardization by an internal reference population of 735 tumors spanning 35 histologies as follows: rank values “Low” (0–24), “Intermediate” (25–74), and “High” (75–100).

The median rank values of T-cell priming markers RNA expression ranged from 24.5 (IFNG) to 63 (ICOSLG). The ranges of rank values of RNA expression for all T-cell priming markers were 0–100 or 0–99. The proportions of patients with low expression (RNA expression rank value: 0–24) of each T-cell priming marker ranged from 14.7% (ICOSLG) to 50.0% (IFNG). The proportions of those with intermediate expression (RNA expression rank value: 25–74) ranged from 40.1% (IFNG) to 55.6% (OX40). The genes that showed high expression (RNA expression rank value: 75–100) included ICOSLG (37.4% of patients), followed by OX40 (23.7%) and OX40LG (23.2%). In contrast, IFNG, ICOS, and CD137 less commonly showed high expression (9.9%, 13.6%, and 15.0% of patients, respectively) (Table 1, Fig. 1b).

**Table 1:** RNA expression of T-cell priming markers among diverse cancers (*N* = 514) (see Supplemental Table 1 for functions).

T-cell priming markers	Mean RNA expression (range)^a^	Low RNA expression *N* (%)^a^	Intermediate RNA expression *N* (%)^a^	High RNA expression *N* (%)^a^
CD27	45.2 (0–99)	150 (29.1%)	266 (51.8%)	98 (19.1%)
CD28	43.4 (0–100)	154 (30.0%)	258 (50.2%)	102 (19.8%)
CD40	46.1 (0–100)	153 (29.8%)	247 (48.1%)	114 (22.2%)
CD40LG	43.4 (0–100)	164 (31.9%)	246 (47.9%)	104 (20.2%)
CD80	48.4 (0–99)	126 (24.5%)	273 (53.1%)	115 (22.4%)
CD86	43.0 (0–99)	154 (30.0%)	276 (53.7%)	84 (16.3%)
CD137	41.1 (0–99)	169 (32.9%)	268 (52.1%)	77 (15.0%)
GITR	45.9 (0–99)	147 (28.6%)	268 (52.1%)	99 (19.3%)
GZMB	42.2 (0–99)	178 (34.6%)	251 (48.8%)	85 (16.5%)
ICOS	35.4 (0–99)	226 (44.0%)	218 (42.4%)	70 (13.6%)
ICOSLG	59.7 (0–100)	76 (14.8%)	246 (47.9%)	192 (37.4%)
IFNG	29.9 (0–100)	257 (50.0%)	206 (40.1%)	51 (9.9%)
OX40	50.6 (0–100)	106 (20.6%)	286 (55.6%)	122 (23.7%)
OX40LG	44.4 (0–99)	170 (33.1%)	225 (43.8%)	119 (23.2%)
TBX21	41.7 (0–99)	183 (35.6%)	244 (47.5%)	87 (16.9%)

The expression profiles were stratified by rank values into “Low” (0–24), “Intermediate” (25–74), and “High” (75–100) percentile.

Percentage indicates percent of patients with that rank, e.g., 29.1% of patients showed low RNA expression of CD27 in their tumors

See Methods as well.

*CD* Cluster of differentiation, *GITR* Glucocorticoid-Induced TNFR-Related, *GZMB* Granzyme B, *ICOS* Inducible T Cell Costimulator, *IFNG* Interferon-gamma, *LG* ligand, *TBX* T-Box Transcription Factor.

^a^Transcript abundance was normalized to internal housekeeping gene profiles and ranked (0–100) to standardized by internal a reference population of 735 tumors spanning 35 histologies.

### 97.7% of patients had distinct T cell priming expression patterns

Among 514 patients, 502 (97.7%) had distinct patterns of expression of the 15 different T-cell priming markers interrogated. There were only six identical patterns of RNA expression shared by more than one patient. (Each of the six patterns was shared by only two patients: 12 patients in total).

### T-cell priming marker expression patterns were not correlated with cancer histology

**Fig. 2:**
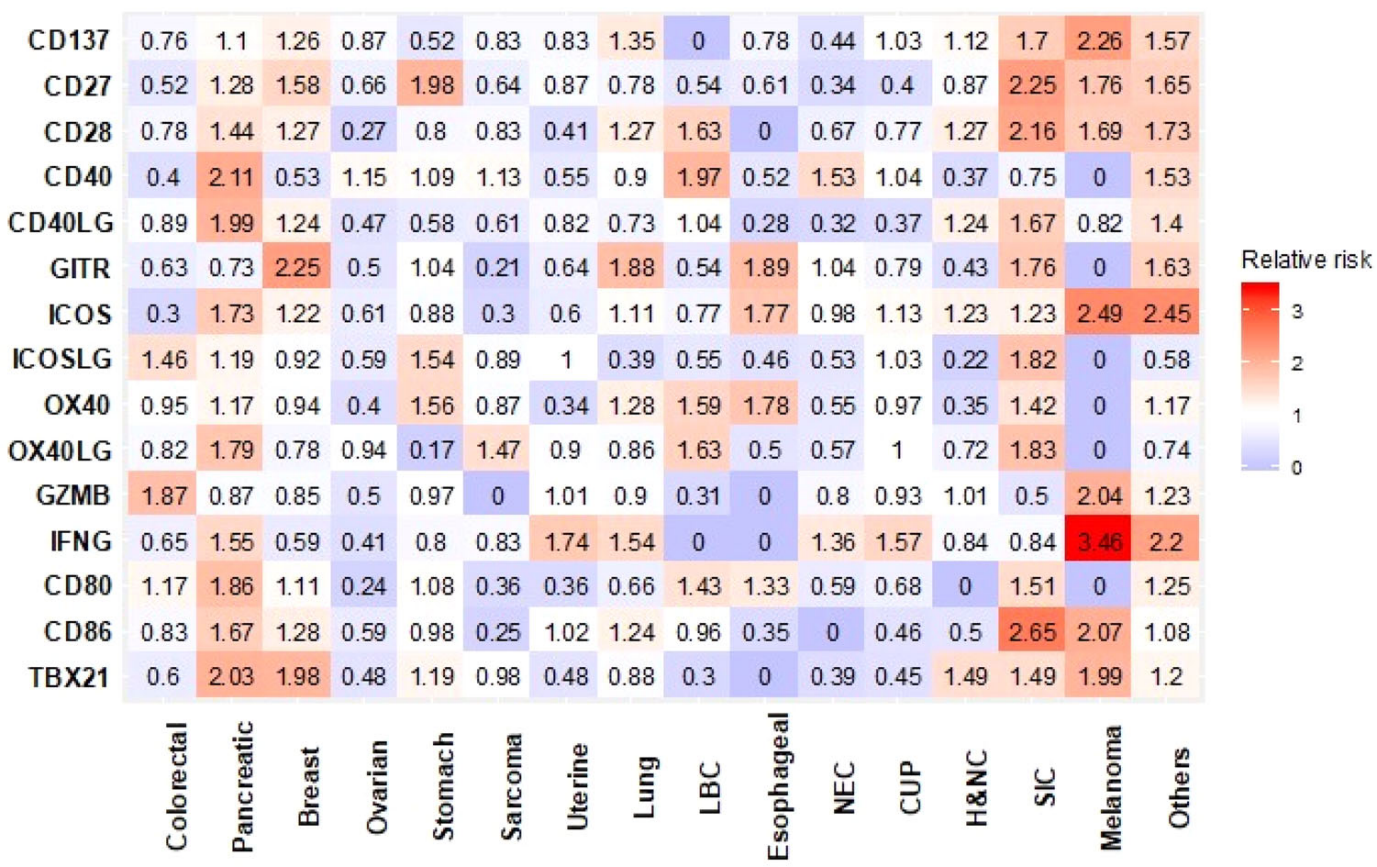
Relative risk of having high RNA expression of T cell priming markers among different types of cancer (*N* = 514). Relative risk compared with all other types of cancer is demonstrated. Red represents higher risk and blue represents lower risk of having high RNA expression. After Bonferroni correction, no significant differences were detected between cancers. Significant *p* value was defined as 0.0002 (0.05/240 variables from T-cell priming markers) or less after Bonferroni correction. *P* values were calculated with chi square test. Others include: cervical cancer (*N* = 5), bladder cancer (*N* = 4), gallbladder and extrahepatic bile duct cancers (*N* = 4), prostate cancer (*N* = 4), brain and nervous system cancers (*N* = 3), kidney and renal pelvis cancers (*N* = 3), squamous cell carcinoma of the skin (*N* = 3), thyroid cancer (*N* = 3), adrenal gland cancer (*N* = 3), lipomatous neoplasm (*N* = 2), mesothelioma (*N* = 2), basal cell carcinoma of the skin (*N* = 1), ocular melanoma (*N* = 1), primary peritoneal carcinoma (*N* = 1), and thymic cancer (*N* = 1). CUP cancer of unknown primary, H&NC head and neck cancer, LBC liver and bile duct cancer, NEC neuroendocrine cancer, SIC small intestine cancer.

**Fig. 3:**
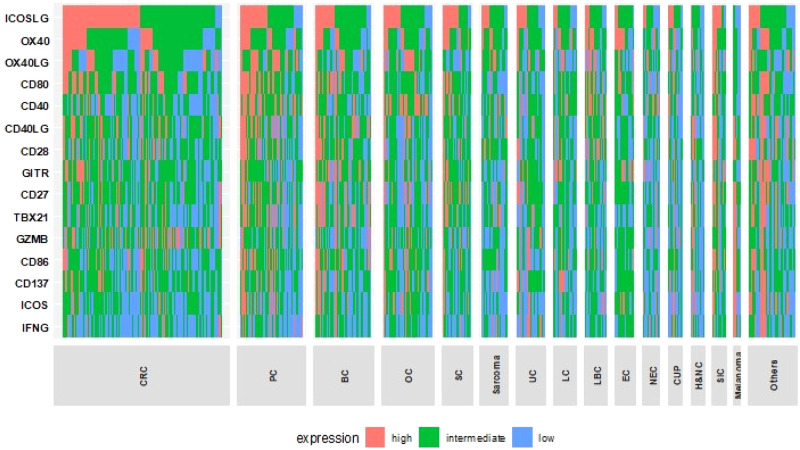
Expression profile of T-cell priming markers in various types of cancer (*N* = 514). Transcript abundance was normalized to internal housekeeping gene profiles and ranked (0–100) to standardized values by comparing to a reference population of 735 tumors spanning 35 histologies. This figure shows no pattern of expression that can be differentiated by tumor type. The expression profiles were stratified by rank values into “Low” (0–24), “Intermediate” (25–74), and “High” (75–100). See *Methods as well.* Colorectal (*N* = 140), pancreatic (*N* = 55), breast (*N* = 49), ovarian (*N* = 43), stomach (*N* = 25), sarcoma (*N* = 24), uterine (*N* = 24), lung (*N* = 20), liver and bile Duct (*N* = 19), esophageal (*N* = 17), neuroendocrine (*N* = 15), cancer of unknown primary (*N* = 13), head and neck (*N* = 12), small intentine cancer (*N* = 12), Melanoma (*N* = 6). Others include: cervical cancer (*N* = 5), bladder cancer (*N* = 4), gallbladder and extrahepatic bile duct cancers (*N* = 4), prostate cancer (*N* = 4), brain and nervous system cancers (*N* = 3), kidney and renal pelvis cancers (*N* = 3), squamous cell carcinoma of the skin (*N* = 3), thyroid cancer (*N* = 3), adrenal gland cancer (*N* = 3), lipomatous neoplasm (*N* = 2), mesothelioma (*N* = 2), basal cell carcinoma of the skin (*N* = 1), ocular melanoma (*N* = 1), primary peritoneal carcinoma (*N* = 1), and thymic cancer (*N* = 1). Each column represents a patient. red, green, and blue means high, intermediate and low expression respectively. BC breast cancer, CRC colorectal cancer, CUP cancer of unknown primary, H&NC head and neck cancer, LBC liver and bile duct cancer, LC lung cancer, NEC neuroendocrine cancer, OC ovarian cancer, PC pancreatic cancer, SC stomach cancer, SIC small intestine cancer, UC uterine cancer.

Figure 2 summarize the relative risk of high expression of T-cell priming markers in each histological type of cancer compared with all other types. Variance in T-cell priming marker expression depending on histological types was observed. For instance, patients with colorectal cancer more commonly had high expression of GZMB and ICOSLG (Relative risk [RR]: 1.87 and 1.46, respectively). In contrast, colorectal cancer patients rarely had high expression in ICOS and CD40 (RR: 0.30 and 0.40, respectively). Despite numeric difference seen in RR, after the Bonferroni correction, no statistically significant difference was observed in each T-cell priming marker expression based on cancer types. Moreover, the heatmap showing expression profile of T-cell priming markers did not show specific expression patterns based on histological types. (Fig. 3).

### Patients with unstable MSI, high TMB and high PD-L1 expression had significantly higher expression of GZMB and IFNG

The mean rank values of each T-cell priming marker RNA expression depending on MSI, TMB and PD-L1 were investigated to reveal their association. Patients with MSI-high (*N* = 15) showed significantly higher RNA expression of GZMB and IFNG compared to those with microsatellite stable status (*N* = 425). (Mean rank value: 66.9 vs. 42.5 and 60.5 vs. 29.3, respectively, *p* value: 0.001 and <0.001, respectively, Table 2).

**Table 2:** Expression of T-cell priming markers based on microsatellite status (*N* = 440)^a^, tumor mutational burden (*N* = 450)^b^ and programmed death ligand 1 expression, (*N* = 513)^c^ (See Supplementary Table 1 for functions of T-cell priming markers).

	Microsatellite status			Tumor mutational burden (mutations/mb)			Programmed death ligand 1 expression		
T-cell priming markers	Stable (*N* = 425)^d^	Unstable (*N* = 15)^d^	*P* value	<10 (*N* = 417)^d^	≥10 (*N* = 33)^d^	*P* value	<1% (*N* = 358)^d^	≥1% (*N* = 155)^d^	*P* value
CD27	45.4 (0–99)	60.1 (12–87)	0.051	42.9 (0–99)	45.5 (0–91)	0.61	43.1 (0–99)	50.3 (0–99)	0.008
CD28	43.9 (0–100)	39.7 (13–87)	0.58	41.4 (0–98)	33.9 (0–88)	0.13	44.6 (0–100)	40.8 (0–98)	0.16
CD40	46.3 (0–100)	44.1 (6–82)	0.77	45.1 (0–100)	33.0 (0–86)	0.019	44.2 (0–99)	50.4 (0–100)	0.022
CD40LG	44.3 (0–100)	45.1 (0–78)	0.92	41.2 (0–99)	35.6 (0–88)	0.28	45.1 (0–100)	40.0 (0–99)	0.076
CD80	48.5 (0–99)	62.3 (29–82)	0.057	46.2 (0–99)	50.2 (3–91)	0.42	46.4 (0–98)	53.2 (0–99)	0.010
CD86	42.6 (0–99)	57.5 (12–90)	0.042	40.8 (0–99)	43.2 (0–90)	0.63	41.2 (0–99)	47.3 (0–98)	0.021
CD137	40.8 (0–99)	56.7 (26–79)	0.031	38.4 (0–99)	45.2 (0–95)	0.17	37.5 (0–99)	49.4 (0–99)	<0.001^e^
GITR	46.3 (0–99)	54.1 (27–75)	0.29	45.3 (0–99)	46.2 (0–99)	0.86	42.0 (0–99)	55.3 (0–99)	<0.001^e^
GZMB	42.5 (0–99)	66.9 (6–98)	0.001^e^	39.9 (0–99)	55.5 (1–98)	0.002^e^	38.7 (0–98)	50.8 (0–99)	<0.001^e^
ICOS	36.2 (0–99)	43.9 (0–84)	0.32	33.2 (0–99)	36.5 (0–90)	0.53	32.5 (0–99)	42.4 (0–99)	<0.001^e^
ICOSLG	60.3 (0–99)	58.9 (21–96)	0.85	59.3 (0–100)	57.2 (0–98)	0.69	61.5 (0–100)	55.8 (0–99)	0.072
IFNG	29.3 (0–99)	60.5 (16–93)	<0.001^e^	27.4 (0–100)	44.0 (0–93)	<0.001^e^	25.2 (0–100)	40.5 (0–99)	<0.001^e^
OX40	50.5 (0–100)	59.9 (18–93)	0.18	48.7 (0–100)	51.2 (0–93)	0.60	49.2 (0–100)	53.8 (0–100)	0.10
OX40LG	44.4 (0–99)	44.8 (5–97)	0.96	44.0 (0–99)	35.1 (0–97)	0.11	45.5 (0–99)	41.9 (0–96)	0.22
TBX21	41.7 (0–99)	57.2 (15–80)	0.042	39.1 (0–99)	42.4 (0–99)	0.52	40.6 (0–98)	44.6 (0–99)	0.14

*CD* Cluster of differentiation, *GITR* Glucocorticoid-Induced TNFR-Related, *GZMB* Granzyme B, *ICOS* Inducible T Cell Costimulator, *IFNG* Interferon-gamma, *LG* ligand, *TBX* T-Box Transcription Factor.

^a^440 patients were evaluable for both T-cell priming marker expression and microsatellite status. 74 patients were not evaluated for microsatellite status.

^b^450 patients were evaluable for both T-cell priming marker expression and tumor mutational burden. 64 patients were not evaluated for tumor mutational burden.

^c^513 patients were evaluable for both T-cell priming marker expression and PD-L1 status. One patient had insufficient quality of PD-L1 testing.

^d^Mean and range of rank value of each gene expression are demonstrated. For instance, for patients with microsatellite stable, there are 425 patients and their mean rank value for CD27 expression was 45.4 and the range was 0–99. The rank value is based on the percentile.

^e^Significant *p* value (two-sided *t*-test) was defined as ≤0.003 (0.05/15 variables from T-cell priming markers) after Bonferroni correction. Transcript abundance was normalized to internal housekeeping gene profiles and ranked (0–100), standardized by internal reference population of 735 tumors spanning 35 histologies. See the Methods as well.

TMB ≥ 10 mutations/megabase (*N* = 33) was associated with significantly higher RNA expression of GZMB and IFNG compared to TMB < 10 mutations/megabase (*N* = 417). (Mean rank value: 55.5 vs. 39.9 and 44.0 vs. 27.4, respectively, *p* value: 0.002 and <0.001, respectively, Table 2).

Patients with PD-L1 ≥ 1% (*N* = 146) demonstrated significantly higher RNA expression of GZMB and IFNG relatively to those with PD-L1 < 1% (*N* = 351) as well. (Mean rank value: 50.8 vs. 38.7 and 40.5 vs. 25.2, respectively, *p* value < 0.001 for both, Table 2). In addition, PD-L1 ≥ 1% was associated with significantly higher expression of CD137, GITR and ICOS. (Mean rank value: 49.4 vs. 37.5, 55.3 vs. 42.0 and 42.4 vs. 32.5, respectively, *p* value < 0.001 for all, Table 2).

### Clustering of T-cell priming marker depending on RNA expression pattern into hot, cold and mixed sub-groups

**Fig. 4:**
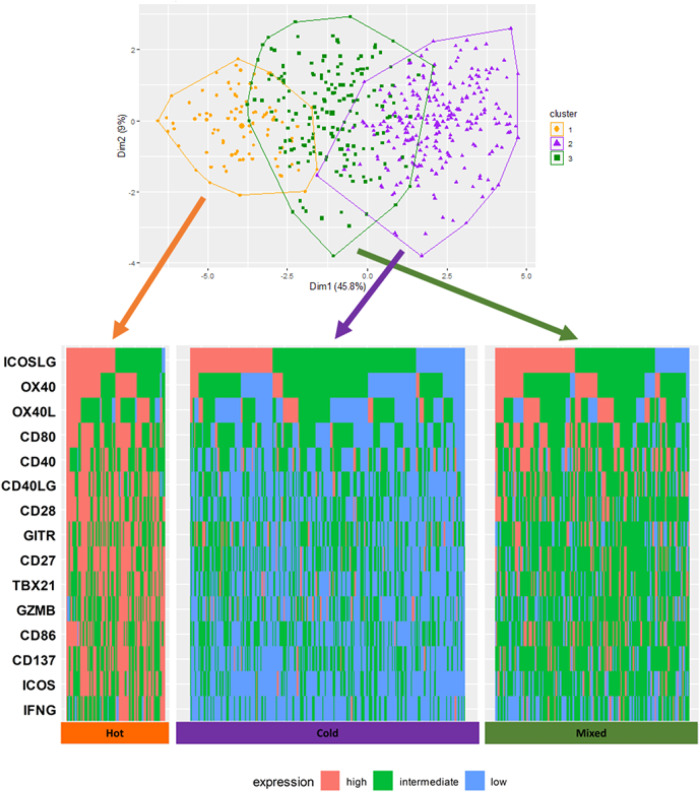
Cluster plot based on T-cell priming marker expression by Ward’s method^30^ (*N* = 514). Principal component analysis was performed and the data points according to the first two principal components that explain the majority of the variance was plotted. Briefly, dimension 1 (Dim 1) represents the value on the vector in the 15-dimensional field, which accounts for the largest possible variance, and it accounts for 45.8% of all variance of the 15 different T-cell priming gene expression in 514 samples. Dimension 2 (Dim 2) is the value on the vector that accounts the second largest possible variance. Dim 2 explains 9% of total variance in the dataset. Ward’s method is a hierarchical clustering method to assign the data points to preset number of clusters to minimize the within-cluster variance. In this analysis, patients were clustered into three clusters. Orange, purple and dark green dots represent the patients classified into cluster 1, 2, and 3, respectively. Hot cluster is one of the three clusters identified by Ward’s hierarchical clustering, which has characteristics of generally high expressions of T-cell priming markers. Cold cluster is one of the three clusters identified by Ward’s hierarchical clustering, which has characteristics of generally low expressions of T-cell priming markers. Mixed cluster is one of the three clusters identified by Ward’s hierarchical clustering, which has characteristics of mixed expression levels of T-cell priming markers. Transcript abundance was normalized to internal housekeeping gene profiles and ranked (0–100) to standardization by an internal reference population of 735 tumors spanning 35 histologies. The expression profiles were stratified by rank values into “Low” (0–24), “Intermediate” (25–74), and “High” (75–100). See *Methods* as well. Each column represents each patient. Red, green, and blue means high, intermediate, and low expression respectively. According to the silhouette method based on T-cell priming marker RNA expression, the optimal number of clusters in this dataset was three: Hot” (cluster 1, with high expression of most T-cell priming markers, *N* = 78), “Cold” (cluster 2, with low expression of most T-cell priming markers, *N* = 210) and “Mixed” (cluster 3, anything not classified into “Hot” or “Cold”, *N* = 137).

Before clustering, the correlations of each T-cell priming marker expression were analyzed to anticipate the expression patterns through hierarchical clustering. Significant positive correlations in most T-cell priming markers were observed. (Supplementary Fig. 1) According to the 30 different indices to determine the best number of clusters, based on T-cell priming marker RNA expression, the optimal number of clusters in this dataset was three. (Supplementary Fig. 2) The samples were classified into three clusters by Ward’s method^30^. (See Methods and Fig. 4, visualized by principal component analysis) Each cluster had characteristic expression patterns of T-cell priming markers—“Hot” (cluster 1, with high expression of most T-cell priming markers, *N* = 78), “Cold” (cluster 2, with low expression of most T-cell priming markers, *N* = 210) and “Mixed” (cluster 3, anything not classified into “Hot” or “Cold”, *N* = 137). (Fig. 4).

### Hot clusters (high expression of most T-cell priming markers) correlated with PD-L1 expression, but not with MSI, TMB or histologic type

The clusters were associated with PD-L1 status. While the proportion of patients with PD-L1 ≥ 1% was 41.0% and 42.3% in hot and mixed cluster, respectively, only 26.7% of patients in Cold were PD-L1 ≥ 1%. (*p* = 0.0043) Although it was not statistically significant, patients in cold cluster rarely showed MSI unstable. (1.5% vs. 6.6% in hot cluster) There was no statistically significant difference among the clusters in terms of the proportion of high TMB or histological types of cancer. (Table 3).

**Table 3:** Characteristics of hot, cold and mixed clusters (*N* = 514 for T-cell priming marker expression; *N* = 388 for other variables including histologies, MSI, TMB and PD-L1 status) (see Supplemental Table 1 for T-cell priming marker functions)^a^.

T-cell priming markers	Hot cluster (*N* = 89)^b^ high expression *N* (%)	Cold cluster (*N* = 249)^c^ high expression *N* (%)	Mixed cluster (*N* = 176)^d^ high expression *N* (%)	
CD27	61 (68.5%)	8 (3.2%)	29 (16.5%)	
CD28	58 (65.2%)	4 (1.6%)	40 (22.7%)	
CD40	36 (40.4%)	21 (8.4%)	57 (32.4%)	
CD40LG	53 (60.0%)	7 (2.8%)	44 (25.0%)	
CD80	54 (60.7%)	10 (4.0%)	51 (29.0%)	
CD86	47 (52.8%)	1 (0.4%)	36 (20.5%)	
CD137	48 (53.9%)	5 (2.0%)	24 (13.6%)	
GITR	46 (51.7%)	25 (10.0%)	28 (15.9%)	
GZMB	45 (50.6%)	20 (8.0%)	20 (11.4%)	
ICOS	50 (56.2%)	3 (1.2%)	17 (9.7%)	
ICOSLG	44 (49.4%)	75 (30.1%)	73 (41.5%)	
IFNG	34 (38.2%)	3 (1.2%)	14 (8.0%)	
OX40	51 (57.3%)	20 (8.0%)	51 (29.0%)	
OX40LG	32 (36.0%)	33 (13.3%)	54 (30.7%)	
TBX21	52 (58.4%)	9 (3.6%)	26 (14.8%)	

Other variables	Hot cluster (*N* = 61) *N* (%)	Cold cluster (*N* = 203) *N* (%)	Mixed cluster (*N* = 124) *N* (%)	*P* value
MSI Unstable	4 (6.6%)	3 (1.5%)	7 (5.6%)	0.059
TMB ≥ 10 mutations/mb	6 (9.8%)	15 (7.4%)	10 (8.1%)	0.83
PD-L1 ≥ 1%	27 (44.3%)	44 (21.7%)	50 (40.3%)	<0.001^e^
Colorectal Cancer	15 (24.6%)	62 (30.5%)	34 (27.4%)	0.94
Pancreatic Cancer	8 (13.1%)	14 (6.9%)	15 (12.1%)	0.17
Breast Cancer	9 (14.8%)	20 (9.9%)	11 (8.9%)	0.44

For the genes, the numbers and percent of patients in each cluster with high expression are demonstrated. For other variables, the number and percentages of patients in each cluster meeting each criterion are demonstrated. For instance, 48 patients (53.9%) in Hot cluster have high expression of CD137; high expression is defined as rank value ≥75th percentile. 4 patients (6.6%) in Hot cluster had MSI unstable status.

*CD* Cluster of differentiation, *GITR* Glucocorticoid-Induced TNFR-Related, *GZMB* Granzyme B, *ICOS* Inducible T Cell Costimulator, *IFNG* Interferon-gamma, *LG* ligand, *MSI* microsatellite instability, *PD-L1* programmed death ligand 1, *TBX* T-Box Transcription Factor, *TMB* tumor mutational burden.

^a^514 patients were evaluated for T-cell priming marker expression; 388/514 had all data of T-cell priming marker expression, MSI status, TMB, and PD-L1 status.

^b^Hot cluster is one of the three clusters identified by Ward’s hierarchical clustering, and has characteristics of generally high expression of T-cell priming markers.

^c^Cold cluster has characteristics of generally low expressions of T-cell priming markers.

^d^Mixed cluster has characteristics of mixed expression levels of T-cell priming markers.

^e^Significant *p* value (chi square test) was defined ≤0.008 (0.05/6 variables) for other variables.

### Tissue of origin of did not correlate with T-cell priming marker expression

To test the value of tissue stratification for separating samples by T-cell priming marker expression, we compared the silhouette scores of clusters formed by histologies before and after histologies are randomized. If histologies and T-cell priming marker expression patterns are connected, we expect the randomization of histologies to reduce the silhouette score of clusters formed by histologies. Our results do not support this hypothesis: a mean silhouette score of −0.0958 was obtained when considering large clusters (i.e., larger than median cluster size, *N* = 40, Supplementary Fig. 3). In comparison, 100,000 randomizations of histologies resulted in a silhouette score of −0.105 ± 0.017 (mean and standard deviation). Our results thus indicate that tissue of origin randomization resulted in improved, not degraded, silhouette scores.

## Discussion

In this analysis, the diversity of 15 T-cell priming marker transcriptomic patterns across multiple types of advanced cancers in 514 patients was demonstrated. Overall, 97.7% of tumors had unique expression patterns of the T-cell priming markers, not seen in any other patient. Similarly, Derks et al., by evaluating histology and RNA expression, previously reported heterogeneity of immune phenotypes of gastroesophageal adenocarcinoma^31^. They discussed that this heterogeneity may explain poor responses to ICB in gastroesophageal adenocarcinoma^32^. Heterogeneity of the immune microenvironment has also been observed in lung^33^, ovarian^34^, breast^35^, and nasopharyngeal cancer^36^, and it may influence response to immunotherapy and prognosis. The diversity of T-cell priming marker expression pattern that we describe herein further supports the concept of immunogram heterogeneity previously noted by other groups in individual histologies and with other types of immune markers. In particular, T-cell priming markers showed no clustering within histology/organ of origin cancer type analysis.

Interestingly we found that GZMB was highly expressed in patients with unstable MSI, high TMB, and high PD-L1 expression. GZMB is one of the most abundant serine proteases stored in secretion granules of activated T cells and NK cells. In the tumor microenvironment, secreted GZMB enters cancer cells by a perforin-dependent mechanism and activates cascades leading to apoptosis^37^. Larimer et al. reported higher expression of GZMB in melanoma patients who responded to ICB compared to non-responders^38^. The correlation between higher expression of GZMB and unstable MSI has also been observed in colorectal cancer^39^, endometrial cancer^40^, and gastric cancer^41^. There has been no prior study regarding the correlation between TMB and expression of GZMB, but a retrospective analysis on lung adenocarcinoma showed that both TMB and RNA expression of GZMB are decreased substantially with age^42^. In high-grade serous ovarian cancer, higher expression of PD-L1 is associated with higher expression of GZMB RNA^43^, a result similar to that seen in our dataset, albeit across tumor types. The result of the current study therefore reinforces a cancer immunity role for GZMB in MSI, TMB high or PD-L1 high tumors.

We also noted that RNA expression of IFNG was significantly higher in patients with unstable MSI, high TMB, and high PD-L1 expression. IFNG is one of the cytokines secreted by NK cells, activated T cells, and antigen-presenting cells (APCs). IFNG demonstrates an anti-tumor effect by activating cytotoxic T cells and inducing tumoricidal effect by APCs, but it also promotes a state of adaptive resistance caused by the upregulation of inhibitory molecules^44^. The correlation between high IFNG expression and unstable MSI has been reported in colon cancer^45^. As seen in GZMB, RNA expression of IFNG is higher in younger patients, who have higher TMB than older patients, suggesting the correlation between IFNG expression and TMB^42^. It has been reported that PD-L1 expression is induced by IFNG, leading to immune tolerance^46,47^. The finding of significantly higher IFNG RNA expression in unstable MSI, high TMB, and high PD-L1 expression is compatible with previous reports and adds a pan-cancer landscape.

We additionally found that CD137, GITR, and ICOS showed significantly higher expression in patients with high PD-L1 expression. CD137 is mostly expressed on activated T cells or NK cells and the binding of CD137 ligand to CD 137 activates T cells leading to an anti-cancer effect^48^. It has been reported that CD137 signaling induces the production of INFG in T cells, which can lead to higher expression of PD-L1^49^. GITR is expressed on regulatory, naïve, and memory T cells and binding of GITR to its ligand leads to immune-stimulatory signals^50^. In breast cancer, significantly higher GITR expression on tumor-infiltrating lymphocytes is seen in patients with positive PD-L1^51^. ICOS is expressed in activated T cells, and its ligand is expressed by B cells and APCs. ICOS signaling induces immune stimulation^52^. It has been reported that ICOS expression is enhanced after anti-PD-1 therapy in mice^53^. Since our dataset does not contain the information of previous treatment, further assessment is required to investigate the association between ICB and ICOS expression.

To understand overall patterns of T-cell priming marker expression rather than analyzing each marker separately, we conducted hierarchical clustering with Ward’s method^30^ and classified the T-cell priming marker expression patterns into three clusters. The clusters were significantly associated with PD-L1 status; in particular, the hot cluster (high expression patterns of T-cell priming markers) associated with PD-L1 positivity. The interaction between T-cell priming markers and PD-L1 is complex and not clearly understood. One potential explanation is that a relatively low concentration of inflammatory cytokines, including IFNG, in patients in the cold cluster leads to lower PD-L1 expression and vice versa for the hot cluster^47^. Although it was not statistically significant, patients in the cold cluster rarely had unstable MSI (1.5% vs. 6.6% in the hot cluster). It was previously reported that MSI status is correlated with the intratumoral immune microenvironment, including the number of infiltrating CD8 T cells in colon cancer^54^. The most common type of cancer included in the current analysis was colorectal cancer, which may explain this numerical difference in MSI unstable depending on the clusters based on T-cell priming marker expression patterns (though this did not reach statistical significance). However, there was no significant association between clusters and histological type of cancer, suggesting that histology is not a determinant factor of the immune microenvironment of cancer.

There are several limitations to the described study. Unlike single cell RNA sequencing or immunohistochemistry, bulk RNA sequencing in this analysis does not allow assessment of which cells produce each marker. For example, there are B cells secreting GZMB in the tumor microenvironment in colorectal, breast, prostate, and cervical cancer, and their role in anti-tumor immunity is different from that of T cells^55^. We discussed the potential explanation for the association between high GZMB expression with unstable MSI, high TMB, and high PD-L1 expression based on the assumption that most GZMB expression is from cytotoxic cells, but the influence from GZMB-secreting B cells cannot be excluded. Therefore, further analysis specifying the cell types responsible for each RNA expression is warranted. In addition, various cancer types were analyzed in a combined manner rather than separately so that a pan-cancer perspective is incorporated into the anti-cancer immunome. However, there is a histology-specific finding in cancer immunity as well. For instance, the association between unstable MSI and increased number of infiltrating CD8 T cells is only observed in colorectal cancer, but not in endometrial carcinoma^54^. Detailed analysis focusing on each histological type of cancer is also required for a better understanding of anti-tumor immunity. The understanding of the role of T-cell priming markers is still in early stages. It is thought that CD80, one of the T-cell priming markers, binds to CD28 on active T cells to activate immune-stimulating signals^56^, so the agonist of CD28 was used in a clinical trial, which resulted in severe cytokine storm in healthy participants^57^. It is also known that CTLA-4 is upregulated through CD80/CD86 and CD28 interaction, causing peripheral immune tolerance^58^. Based on this finding, galiximab, an anti-CD80 antibody, was studied as a treatment of B cell lymphoma^59^. The fact that both agonist and antagonist of the same signaling pathway were studied to treat cancer suggests a limited understanding of the mechanism of cancer immunity. It is also estimated that more genes than the 15 markers analyzed in this analysis play a role in T-cell priming. The effort to integrate large multi-omics data has been promising to detect genes involved in cancer immunity^60,61^. The finding of heterogeneity in T-cell priming marker expression needs to be interrogated when a new gene with T-cell priming role is discovered through such effort. Future analyses should also correlate TPM expression with that of additional immune markers. In addition, validation of the finding in additional cohorts of patients with advanced or metastatic disease will be necessary. Another limitation of the study is that patients may have had a variety of prior therapies. Our study does not include clinical correlations, which will be important for future work. Further research on the molecular biology regarding cancer immunity is necessary for better design of future clinical trials targeting T-cell priming markers. Future studies should try to establish the individual contribution of TPMs, as well as other immune markers to outcome.

Despite several limitations, to the best of our knowledge, this is one of the first pan-cancer transcriptome analyses of T-cell priming markers. There are more studies analyzing signatures of cancer immunity comprehensively using larger cohorts^12,13^, but no other study has focused on T-cell priming marker as exclusively as this study. The finding of the heterogeneity of T-cell priming marker expression pattern unrelated to histological types of cancer may explain the limited response rate in previous clinical trials targeting T-cell priming markers. We may be in a similar situation as the early developmental phase of targeted therapy for cancer with a specific genomic mutation. For instance, gefitinib, one of the EGFR inhibitors, was initially investigated in a clinical trial for patients with advanced non-small cell lung cancer regardless of *EGFR* mutation status, showing a response rate of 9–12%^62^. However, in a subsequent trial with an inclusion criterion of *EGFR* mutation, the response rate was increased above 70%^63^. Hence, we may need to select patients for specific immunotherapies based on the immunomic state in the tumor microenvironment in order to maximize treatment effects in clinical trials targeting T-cell priming markers, as a recent clinical trial demonstrated a feasibility and efficacy of patient selection based on tumor molecular subtypes in metastatic clear cell renal carcinoma^29^.

In conclusion, the transcriptome of T-cell priming markers showed immunomic heterogeneity across different histological types of cancer. Furthermore, almost all tumors had a T-cell priming immunogram that was complex and also distinct from other tumors. Therefore, in order optimize efficacy of agents targeting T cell priming, selection of the patients based on immunomic state of the tumor microenvironment, rather than histologic type of cancer or other factors may be needed.

## Methods

### Patients

The RNA expression levels of 15 T-cell priming markers in various types of solid tumors from 514 patients seen at the University of California San Diego (UCSD) Moores Cancer Center for Personalized Therapy were analyzed at a Clinical Laboratory Improvement Amendments (CLIA)-licensed and College of American Pathologist (CAP)-accredited clinical laboratory, OmniSeq (https://www.omniseq.com/). Normally, the patients with advanced cancer who exhaust standard treatments are referred to the UCSD Moores Cancer Center for Personalized Therapy. All patients in the clinic who consented to participate in this observational study were included, regardless of their age, sex, race, type and of cancer, previous treatments, or comorbid conditions. In addition to the expression data, the information on the patients’ age, sex, histological types of primary cancer, microsatellite instability (MSI), tumor mutational burden (TMB), and programmed death-ligand 1 (PD-L1) status were collected. If a patient had two or more different samples that were analyzed in different days, the one from earlier timepoint was used for the analysis, which may or may not be a sample from initial diagnosis. The patients included in this analysis provided written informed consent.

### Sampling of tissue and analysis of cancer immunity markers

After the collection, tumors were provided as formalin-fixed, paraffin-embedded (FFPE) samples, and evaluated by RNA sequence at OmniSeq laboratory. All RNA was extracted from FFPE using truXTRAC FFPE extraction kit (Covaris, Inc., Woburn, MA), with some modification to the instruction by the manufacturer. After purification, RNA was dissolved in 50 µL water and the yield was measured through Quant-iT RNA HS assay (Thermo Fisher Scientific, Waltham, MA), as per the manufacturer’s recommendation. For appropriate library preparation, the pre-defined titer of 10 ng RNA was referred to as acceptance criteria. Torrent Suite’s plugin immuneResponseRNA (v5.2.0.0) 34 was used for the absolute reading of the RNA sequence. The RNA expression of 397 different genes was measured. Among them, we focused on 15 genes that are related to T cell priming related cancer immunity, including CD27, CD28, CD80, CD86, CD40, CD40LG, CD137, GITR, GZMB, ICOS, ICOSLG, IFNG, OX40, OX40LG, and TBX21. (T-cell priming markers, Supplementary Table 1 and Table 2).

Transcript abundance was normalized to internal housekeeping gene profiles and ranked (0–100 percentile) in a standardized manner to a reference population of 735 tumors spanning 35 histologies. The expression profiles were stratified by rank values into “Low” (0–24), “Intermediate” (25–74), and “High” (75–100) to categorize expression level of each marker and analyze the similarities of T-cell priming marker expression patterns among patients in a clear-cut manner^64^.

### Definitions and measurements of MSI, TMB and PD-L1 expression

As for MSI, genomic DNA was extracted from qualified FFPE tumors (>20% neoplastic nuclei) by means of the truXTRAC FFPE extraction kit (Covaris). The MSI-NGS assay evaluates 29 homopolymer loci, including BAT-25 and BAT-26, by sequencing tumor DNA (20 ng) on an Illumina MiSeq Sequencer. The assay has a computational tool, MSI-NGS Caller, which compares the tumor homopolymer repeat profile of a sample to a normal allele distribution predefined at each locus, to make MSI calls (Unstable, Stable or Inconclusive) without the need for a matched normal DNA^65^.

For TMB, genomic DNA was extracted from qualified FFPE tumors (>30% neoplastic nuclei) by means of the truXTRAC FFPE extraction kit (Covaris) with 10 ng DNA input for library preparation. DNA Libraries were prepared with Ion AmpliSeq targeted sequencing chemistry using the Comprehensive Cancer Panel, followed by enrichment and template preparation using the Ion Chef system, and sequencing on the Ion S5XL 540 chip (Thermo Fisher Scientific). Following removal of germline variants, synonymous variants, indels and SNVs with <5% VAF, TMB is reported as eligible mutations per qualified panel size (Mutations/Megabase)^64^. TMB ≥ 10 was set as a cut off since FDA has approved pembrolizumab for advanced cancer with high TMB, based on the finding of KEYNOTE-158 trial, which used TMB ≥ 10 as a cutoff^66^.

The measurement of PD-L1 was conducted by three different immunohistochemistry (IHC) assays; Dako PD-L1 IHC 22C3 pharmDx assay, Dako PD-L1 IHC 28-8 pharmDx assay (Dako North America, Inc., Carpinteria, California, USA, *N* = 474, 6, respectively), and VENTANA PD-L1 (SP142) assay (Ventana Medical Systems, Inc., Tuscon, Arizona, USA, *N* = 33). The cutoff of 1% was used in the analysis since 1% is the minimal expression of PD-L1 with clinical significance^67^.

### Clustering and statistical methods

Statistical analysis was verified by our statistician/bioinformatician (ER). Patient’s baseline characteristics and the frequency of T-cell priming markers were summarized by descriptive statistics. All statistical analyses were conducted with R 3.6.1 (R Foundation for Statistical Computing, Vienna, Austria). Hierarchical clustering was conducted to classify the samples into distinct groups based on expression patterns of T-cell priming markers. The optimal number of clusters was estimated by 30 different indices using R package “NbClust”^68^. Ward’s minimum variance method was utilized for cluster production^30^. The similarity of each sample’s T-cell priming markers expression were visualized on the two-dimensional field using principal component analysis^69^. Briefly, dimension 1 (Dim 1) represents the value on the vector which accounts for the largest possible variance, and dimension 2 (Dim 2) is the value on the vector that accounts the second largest possible variance. To quantitively test the connection of histologies to T-cell priming marker expression, the silhouette coefficient (also known as silhouette score) was calculated assigning each sample to cluster based on its histologically determined tissue of origin. Small cluster (i.e., clusters smaller than the median cluster size) were omitted, resulting in removal of 40 samples (of a total of 514). A specialized code, which is available upon request, was developed in the R language, that collects the mean score for 100,000 randomizations of tissue of origin.

R packages “tidyverse”, “cluster”, “factoextra” and “dendextend” were used for these analyses. *P* values were calculated by chi-square test for categorical values. For continuous values, two-sided *t*-test was used to calculate *p* values. Statistical significance was determined by *p* ≤ 0.05 with Bonferroni correction for multiple comparisons.

### Ethical approval and consent to participate

Every investigation was conducted following the guidelines of the UCSD Institutional Review Board for data collection (Study of Personalized Cancer Therapy to Determine Response and Toxicity, UCSD_PREDICT, NCT02478931) and any investigational therapies for which patients consented.

### Reporting summary

Further information on research design is available in the Nature Research Reporting Summary linked to this article.

### Supplementary information

Reporting Summary

Supplementary data

Supplementary figures and tables

## Data Availability

The data analyzed was the processed data from the company OmniSeq. They standardize the expression level of each gene based on their internal reference and provide the percentile of the expression level, which we analyzed here. Therefore, we do not have raw sequencing data. The data are available as Supplementary data.

## Code availability

R packages “NbClust”, “tidyverse”, “cluster”, “factoextra” and “dendextend” were used for these analyses. The codes used and/or analyzed during the current study are available from the corresponding authors on reasonable request.
