# Supplementary material for: T-cell priming transcriptomic markers: implications of immunome heterogeneity for precision immunotherapy
Source: NPJ Genom Med. 2023 Aug 8;8:19. doi: 10.1038/s41525-023-00359-8 (PMC10409760; doi:10.1038/s41525-023-00359-8)
Supplement: Supplementary file 1 — Reporting Summary [file 41525_2023_359_MOESM1_ESM.pdf]

nature portfolio

Corresponding author(s): Hirotaka Miyashita
Shumei Kato

Last updated by author(s): May 6, 2023

Reporting Summary

Nature Portfolio wishes to improve the reproducibility of the work that we publish. This form provides structure for consistency and transparency in reporting. For further information on Nature Portfolio policies, see our Editorial Policies and the Editorial Policy Checklist.

Statistics

For all statistical analyses, confirm that the following items are present in the figure legend, table legend, main text, or Methods section.

n/a	Confirmed	
☐	☒	The exact sample size (*n*) for each experimental group/condition, given as a discrete number and unit of measurement
☐	☒	A statement on whether measurements were taken from distinct samples or whether the same sample was measured repeatedly
☐	☒	The statistical test(s) used AND whether they are one- or two-sided<br>*Only common tests should be described solely by name; describe more complex techniques in the Methods section.*
☐	☒	A description of all covariates tested
☐	☒	A description of any assumptions or corrections, such as tests of normality and adjustment for multiple comparisons
☐	☒	A full description of the statistical parameters including central tendency (e.g. means) or other basic estimates (e.g. regression coefficient) AND variation (e.g. standard deviation) or associated estimates of uncertainty (e.g. confidence intervals)
☐	☒	For null hypothesis testing, the test statistic (e.g. *F*, *t*, *r*) with confidence intervals, effect sizes, degrees of freedom and *P* value noted<br>*Give P values as exact values whenever suitable.*
☒	☐	For Bayesian analysis, information on the choice of priors and Markov chain Monte Carlo settings
☐	☒	For hierarchical and complex designs, identification of the appropriate level for tests and full reporting of outcomes
☒	☐	Estimates of effect sizes (e.g. Cohen's *d*, Pearson's *r*), indicating how they were calculated

Our web collection on statistics for biologists contains articles on many of the points above.

Software and code

Policy information about availability of computer code

Data collection	R 4.0.5, R packages “tidyverse”, “cluster”, “factoextra” and “dendextend” were used for these analyses.
Data analysis	R 4.0.5, R packages “tidyverse”, “cluster”, “factoextra” and “dendextend” were used for these analyses.

For manuscripts utilizing custom algorithms or software that are central to the research but not yet described in published literature, software must be made available to editors and reviewers. We strongly encourage code deposition in a community repository (e.g. GitHub). See the Nature Portfolio guidelines for submitting code & software for further information.

Data

Policy information about availability of data

All manuscripts must include a data availability statement. This statement should provide the following information, where applicable:

- Accession codes, unique identifiers, or web links for publicly available datasets
- A description of any restrictions on data availability
- For clinical datasets or third party data, please ensure that the statement adheres to our policy

The data analyzed was the processed data from the company OmniSeq. They standardize the expression level of each gene based on their internal reference and provide the percentile of the expression level, which we analyzed here. Therefore, we do not have raw sequencing data. The data is available as Supplementary data.

Research involving human participants, their data, or biological material

Policy information about studies with human participants or human data. See also policy information about sex, gender (identity/presentation), and sexual orientation and race, ethnicity and racism.

Reporting on sex and gender	Sixty percent (N = 310) of the patients were women.
Reporting on race, ethnicity, or other socially relevant groupings	No socially relevant groupings were utilized in this study.
Population characteristics	We analyzed 514 samples from patients with a wide variety of advanced/metastatic cancers as summarized in Figure 1a and Supplementary Table 3. The most common type of cancer was colorectal cancer (27.2 %) followed by pancreatic (10.7 %), breast (9.5 %), ovarian (8.4 %) and stomach cancer (4.9 %). The median (range) of the patients' ages was 60.8 years (23.9 to 93.3 years old). Sixty percent (N = 310) of the patients were women.
Recruitment	All patients in the clinic who consented to participate in this observational study were included, regardless of their age, sex, race, type and of cancer, previous treatments, or comorbid conditions.
Ethics oversight	Every investigation was conducted following the guidelines of the UCSD Institutional Review Board for data collection (Study of Personalized Cancer Therapy to Determine Response and Toxicity, UCSD_PREDICT, NCT02478931) and any investigational therapies for which patients consented.

Note that full information on the approval of the study protocol must also be provided in the manuscript.

Field-specific reporting

Please select the one below that is the best fit for your research. If you are not sure, read the appropriate sections before making your selection.

☒ Life sciences ☐ Behavioural & social sciences ☐ Ecological, evolutionary & environmental sciences

For a reference copy of the document with all sections, see nature.com/documents/nr-reporting-summary-flat.pdf

Life sciences study design

All studies must disclose on these points even when the disclosure is negative.

Sample size	All patients in the clinic who consented to participate in this observational study were included, regardless of their age, sex, race, type and of cancer, previous treatments, or comorbid conditions. 514 patients were included.
Data exclusions	If a patient had two or more different samples that were analyzed in different days, the one from earlier timepoint was used for the analysis, which may or may not be a sample from initial diagnosis.
Replication	N/A
Randomization	N/A
Blinding	N/A

Reporting for specific materials, systems and methods

We require information from authors about some types of materials, experimental systems and methods used in many studies. Here, indicate whether each material, system or method listed is relevant to your study. If you are not sure if a list item applies to your research, read the appropriate section before selecting a response.

Materials & experimental systems

n/a	Involved in the study	
☒	☐	Antibodies
☒	☐	Eukaryotic cell lines
☒	☐	Palaeontology and archaeology
☒	☐	Animals and other organisms
☐	☒	Clinical data
☒	☐	Dual use research of concern
☒	☐	Plants

Methods

n/a	Involved in the study	
☒	☐	ChIP-seq
☒	☐	Flow cytometry
☒	☐	MRI-based neuroimaging

Clinical data

Policy information about clinical studies

All manuscripts should comply with the ICMJE guidelines for publication of clinical research and a completed CONSORT checklist must be included with all submissions.

Clinical trial registration	Study of Personalized Cancer Therapy to Determine Response and Toxicity, UCSD_PREDICT, NCT02478931
Study protocol	Study of Personalized Cancer Therapy to Determine Response and Toxicity, UCSD_PREDICT, NCT02478931
Data collection	Every investigation was conducted following the guidelines of the UCSD Institutional Review Board for data collection
Outcomes	We did not assess clinical outcomes in this study.
