# Supplementary material for: T-cell priming transcriptomic markers: implications of immunome heterogeneity for precision immunotherapy
Source: NPJ Genom Med. 2023 Aug 8;8:19. doi: 10.1038/s41525-023-00359-8 (PMC10409760; doi:10.1038/s41525-023-00359-8)
Supplement: Supplementary file 3 — Supplementary figures and tables [file 41525_2023_359_MOESM3_ESM.pdf]

Supplementary Figure 1. Correlation matrix of 15 T-cell priming marker expression

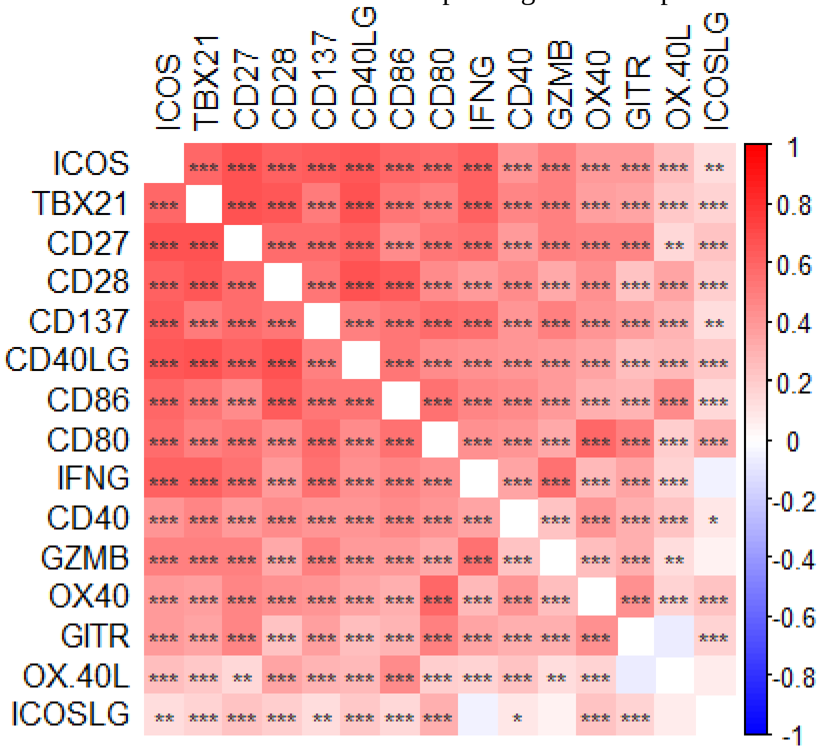

Positive and negative correlation coefficient is described with red and blue, respectively. One, two and three asterisks represent p-value < 0.05, 0.01 and 0.001, respectively. The genes were ordered according to first principal component.

Supplementary Figure 2. Determination of optimal number of clusters through 30 indices

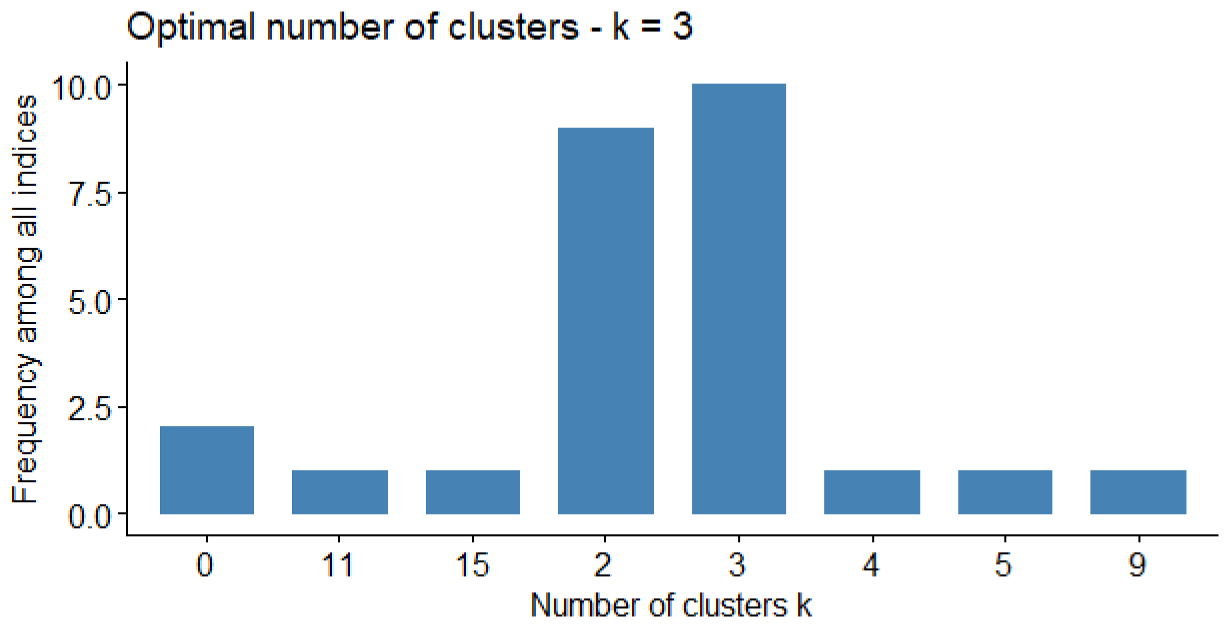

Optimal number of clusters was determined through 30 different indices using R package "NbClust"
The largest number of indices (10 among 30 indices) suggested that three is the optimal number of clusters in the dataset.
The dataset was classified into three different clusters based on this result.

Supplementary Figure 3. Principal component analysis plot with clustering based on histology

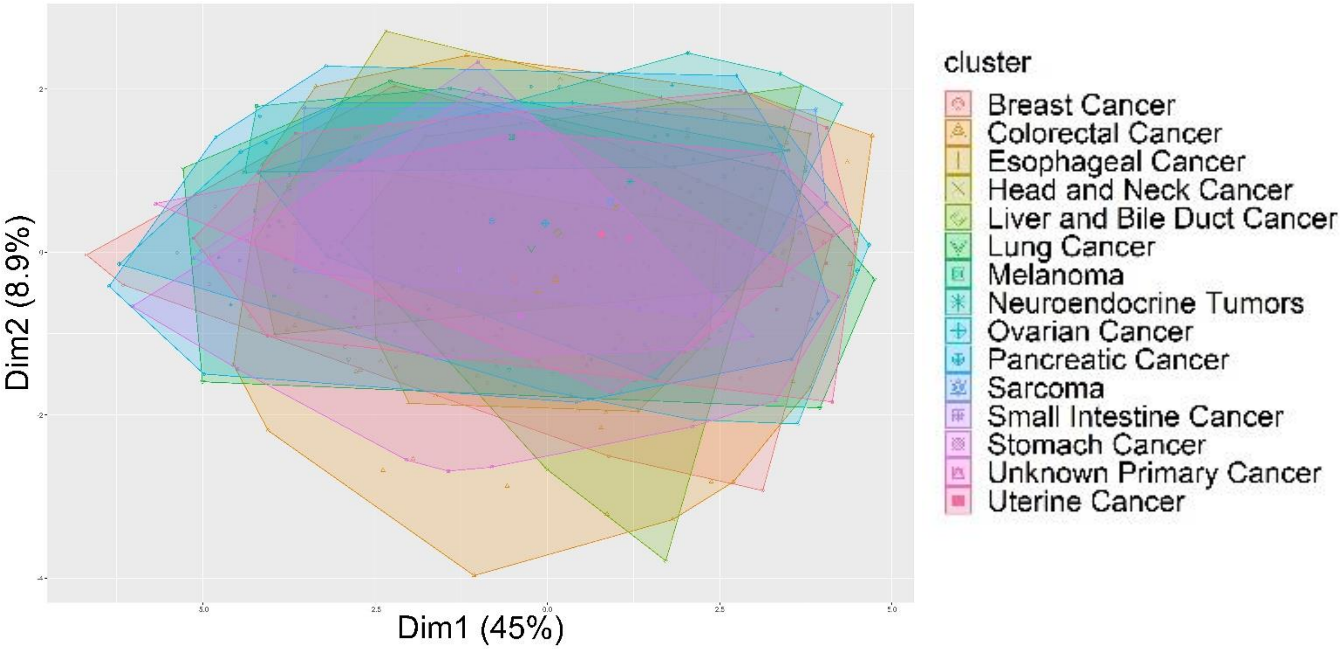

Clustering samples by histologies. The dimension of T-cell priming levels of each sample were reduced to 2 using the Principal Component Analysis method. The 1st and 2nd dimensions are shown, with the fraction of variation in each dimension shown in the corresponding axis. Each tissue is shown in a distinct color, and the "center of gravity" for each cluster is shown as different symbols according to the figure legend. This figure demonstrates that tissue of origin of cancer type did not correlate with T-cell priming marker expression.

Supplementary Table 1: Overview of T-cell priming markers.

**T-cell priming markers**			Supplementary Reference
CD27	CD27 is expressed on naïve and memory T cells.<br>CD70 is the ligand of CD27 and expressed on activated T cells, B cells or APCs.<br>When CD 70 binds to CD27, immune stimulating signals are activated.	Although unclear, CD27 agonists or stimulants may demonstrate anti-tumor effect when CD27 expression is low.<br><br>Potential intervention:<br>Varlilumab:	[1]
CD28	Activated T cells express CD28.<br>CD80 and CD86 are the ligands of CD28 and expressed on APCs.<br>Binding of CD28 to CD80 or CD86 leads to immune stimulating signals.	Tumors with low expression of CD28, CD80 or CD86 may be treated with an agonist or stimulator of this axis.<br><br>Potential intervention:<br>TGN1412 (CD28 agonist. Also known as TAB08)<br>Galiximab (anti-CD80 antibody)	[2]
CD80			
CD86			
CD40	CD40 is expressed on activated T cells. Its ligand, CD40 ligand is expressed on cancer cells, B cells or APCs.<br>When CD40 ligand binds to CD40, immune stimulating signals are activated.	The state of high CD40 expression and low CD40 ligand expression may be targeted by CD40 agonists or stimulators.<br>Potential intervention:<br>ABBV-927<br>ADC-1013<br>ABBV-428	[3]
CD40LG			

CD137 (also known as 4-1BB ligand receptor or TNF Receptor Superfamily Member 9)	CD137 is mostly expressed on activated T cells or NK cells.<br>Cancer cells, antigen presenting cells (APCs) and B cells express CD137 ligand.<br>The binding of CD137 and CD137 ligand brings about immune stimulating signals.	CD137 agonist or stimulator (as below) can possibly show anti-cancer effect.<br><br>Potential intervention (CD137 agonists):<br>Urelumab<br>Utomiluma PRS-343	[4]
GITR (Glucocorticoid-induced tumor necrosis factor receptor)	GITR is expressed on regulatory, naïve and memory T cells.<br>APCs and B cells express the ligand of GITR.<br>Binding of GITR to its ligand leads to immune stimulatory signals.	When GITR expression is high and GITR ligand expression is low, GITR agonists or stimulants can bring about anti-tumor effect.<br><br>Potential intervention:<br>BMS-986156<br>INCAGN01876<br>BMS-986156<br>GWN 323<br>MK-4166	[5]
GZMB (Granzyme B)	Granzyme B is one of the most abundant serine proteases stored in secretion granules of activated T cells and NK cells.<br>Secreted granzyme B activates apoptosis of cancer cells in combination with other granule proteins including perforin.	To our best knowledge, there is no substance targeting GZMB to activate anti-cancer effects.	[6]
ICOS (Inducible T-Cell Costimulator)<br><br>ICOSLG	Activated T cells express ICOS.<br>ICOS ligand is expressed on B cells and APCs.<br>Immune stimulating signals are induced by binding of ICOS to ICOS ligand.	ICOS agonists or stimulants can demonstrate anti-cancer activity in the state with high ICOS expression and low ICOS ligand expression.<br><br>Potential intervention:<br>BMS-986226<br>GSK3359609<br>JTX-2011	[7]

IFNG (Interferon Gamma)	IFNG is one of the cytokines secreted by NK cells, activated T cells and APCs.<br>IFNG demonstrates anti-tumor effect by activating cytotoxic T cells and inducing tumoricidal effect by APCs.<br>Of note, IFNG is reported to induce progression of tumor in a specific context.	An example which targets INFG for anti-cancer effects is as below<br><br>Potential intervention:<br>Emapalumab	[8]
OX40	OX40 is expressed by activated T cells, NK cells, NKT cells and neutrophils.<br>Its ligand, OX40 ligand is expressed by APCs and B cells.	When OX40 expression is high and OX40 ligand expression is low, OX40 agonists or stimulants may lead to anti-cancer effect.<br><br>Potential intervention:<br>ABBV-368<br>BMS-986178<br>GSK3174998<br>INCAGN01949	[9]
OX40LG			
TBX21 (T-Box 21) (also known as T-bet)	TBX21 is a transcription factor expressed in helper T cells.<br>TBX21 regulates the expression of IFNG and controls immune stimulatory signals.	To our best knowledge, there is no active agent under investigation that targets TBX21.	[10]

Abbreviations
APC: antigen presenting cell, CD: Cluster of differentiation, GITR: Glucocorticoid-Induced TNFR-Related, GZMB: Granzyme B, ICOS: Inducible T Cell Costimulator, IFNG: Interferon-gamma, LG: ligand, TBX: T-Box Transcription Factor

Supplementary Table 2: Selected clinical trials targeting T-cell priming markers

Target	Drug	Phase	Type of cancer	Results	Comment	Supplemental Reference
CD27	Varlilumab	I	Various solid tumors (N = 25 and 31 in dose escalation and expansion, respectively)	One patient with metastatic renal cell carcinoma achieved a PR. One patient had dose limiting toxicity – hyponatremia.	Expression of immune markers not required for the enrollment.	[11]
CD28	TGN1412	I	Healthy participants	All of six healthy participants developed cytokine storm.	Trial discontinued.	[12]
CD40	ADC-1013	I	Various solid tumors (N = 23)	Most adverse events were grade 1/2 or transient.	Expression of immune markers not required for the enrollment.	[13]
	ABBV-428	I	Various solid tumors (N = 59)	Grade 3/4 adverse events - pericardial effusion, colitis, infusion-related reaction, and pleural effusion (N = 1 each)<br>36 % (N = 9/25) of patients received recommended phase II dose showed SD.	Expression of immune markers not required for the enrollment.	[14]
CD137	Utomilumab	Ib	Various solid tumors (N = 24)	ORR: 4.2 % (N = 1/24), no dose-limiting toxicity observed.	Expression of immune markers not required for the enrollment.	[15]
	Urelumab	I/II	Resectable pancreatic ductal adenocarcinoma (N = 10 for arm with urelumab)	Neoadjuvant therapy with combination of nivolmab, GM-CSF-secreting allogeneic tumor cell vaccine (GVAX) and urelumab demonstrated 20% (2/10) pathologic response, which was not observed with GVAX alone or GVAX with nivolumab.<br>Nine out of 10 resected patients remain disease free after a median follow up of 12 months.	Expression of immune markers not required for the enrollment.	[16]
		I/II	Various solid tumors (N = 346)	Transaminitis, fatigue and nausea were most common adverse events, seen in 10-20 % of participants.	Expression of immune markers not required for the enrollment.	[17]

					Analysis of three different clinical trials.	
GITR	BMS-986156	I/IIa	Various solid tumors (N = 34 and 258 for monotherapy and combination with nivolumab)	No dose-limiting adverse events observed with monotherapy. One patient receiving combination therapy had grade 4 creatine phosphokinase elevation.<br>ORR: 0 - 11.1 % with combination therapy; no responses with monotherapy.	Expression of immune markers not required for the enrollment.	[18]
ICOS	BMS-986226	I/II	Various solid tumors (planned N = 234)	Clinical outcome not available.	Alone or combination with nivolumab or ipilimumab.<br><br>Expression of immune markers not required for the enrollment.	[19]
OX40	BMS-986178	I/IIa	Various solid tumors (N = 20 and 145 for monotherapy and combination with nivolumab and/or ipilimumab)	Grade 3/4 adverse events occurred in one patient (5%) with monotherapy and 15 patients (10%) with combination.<br>ORR: 0 - 13 % with combination therapy; no responses with monotherapy.	Expression of immune markers not required for the enrollment.	[20]

Abbreviations: CD: Cluster of differentiation, GITR: Glucocorticoid-Induced TNFR-Related, GZMB: Granzyme B, ICOS: Inducible T Cell Costimulator, IFNG: Interferon-gamma, LG: ligand, ORR: objective response rate, PR: partial response; SD: stable disease, TBX: T-Box Transcription Factor

Supplementary Table 3. Demographics of PD-L1, TMB and MSI according to histological types of cancer

Cancer type	MSI Unstable (N,%)[1]	Cancer type	TMB ≥ 10 (N,%)[2]	Cancer type	PD-L1 ≥ 1% (N,%)[3]
Colorectal (N=130)	8 (6%)	Colorectal (N=120)	15 (12.5%)	Colorectal (N=140)	35 (25%)
Pancreatic (N=47)	0 (0%)	Pancreatic (N=43)	0 (0%)	Pancreatic (N=55)	16 (29%)
Breast (N=42)	0 (0%)	Breast (N=46)	1 (2%)	Breast (N=49)	9 (18%)
Ovarian (N=35)	0 (0%)	Ovarian (N=41)	0 (0%)	Ovarian (N=43)	18 (42%)
Stomach (N=19)	2 (11%)	Stomach (N=22)	0 (0%)	Stomach (N=25)	11 (44%)
Sarcoma (N=21)	1 (5%)	Sarcoma (N=23)	1 (4%)	Sarcoma (N=24)	4 (17%)
Uterine (N=21)	2 (10%)	Uterine (N=21)	2 (10%)	Uterine (N=24)	9(38%)
Lung (N=15)	0 (0%)	Lung (N= 17)	3 (18%)	Lung (N=19)	8 (42%)
Liver and Bile Duct (N=14)	0 (0%)	Liver and Bile Duct (N=13)	0 (0%)	Liver and Bile Duct (N=19)	3 (16%)
Esophageal (N=15)	2 (13%)	Esophageal (N=15)	3 (20%)	Esophageal (N=17)	10 (59%)
Neuroendocrine (N=14)	0 (0%)	Neuroendocrine (N=15)	2 (13%)	Neuroendocrine (N=15)	4 (27%)
Unknown primary (N=10)	0 (0%)	Unknown primary (N=12)	2 (17%)	Unknown primary (N=13)	5 (38%)
Head and Neck (N= 11)	0 (0%)	Head and Neck (N=12)	0 (0%)	Head and Neck (N=12)	2 (17%)
Small Intestine (N= 8)	0 (0%)	Small Intestine (N= 9)	1 (11%)	Small Intestine (N=12)	3 (25%)
Melanoma (N=6)	0 (0%)	Melanoma (N=6)	2 (33%)	Melanoma (N=6)	5 (83%)
Others (N=32)	0 (0%)	Others (N=35)	1(3%)	Others (N=40)	13 (33%)

[1] 440 patients were evaluable for both T-cell priming marker expression and microsatellite status. 74 patients were not evaluated for microsatellite status.
[2] 450 patients were evaluable for both T-cell priming marker expression and tumor mutational burden. 64 patients were not evaluated for tumor mutational burden.
[3] 513 patients were evaluable for both T-cell priming marker expression and PD-L1 status. One patient had insufficient quality of PD-L1 testing.

Supplementary References

[1] van de Ven K, Borst J. Targeting the T-cell co-stimulatory CD27/CD70 pathway in cancer immunotherapy: rationale and potential. Immunotherapy 2015;7:655–67. https://doi.org/10.2217/IMT.15.32.

[2] Esensten JH, Helou YA, Chopra G, Weiss A, Bluestone JA. CD28 Costimulation: From Mechanism to Therapy. Immunity 2016;44:973–88. https://doi.org/10.1016/j.immuni.2016.04.020.

[3] Elgueta R, Benson M, VC de V, A W, Y G, RJ N. Molecular mechanism and function of CD40/CD40L engagement in the immune system. Immunol Rev 2009;229:152–72. https://doi.org/10.1111/J.1600-065X.2009.00782.X.

[4] Yonezawa A, Dutt S, Chester C, Kim J, Kohrt HE. Boosting cancer immunotherapy with anti-CD137 antibody therapy. Clin Cancer Res 2015;21:3113–20. https://doi.org/10.1158/1078-0432.CCR-15-0263.

[5] Knee DA, Hewes B, Brogdon JL. Rationale for anti-GITR cancer immunotherapy. Eur J Cancer 2016;67:1–10. https://doi.org/10.1016/j.ejca.2016.06.028.

[6] Martínez-Lostao L, Anel A, Pardo J. How Do Cytotoxic Lymphocytes Kill Cancer Cells? Clin Cancer Res 2015;21:5047–56. https://doi.org/10.1158/1078-0432.CCR-15-0685.

[7] Fan X, Quezada SA, Sepulveda MA, Sharma P, Allison JP. Engagement of the ICOS pathway markedly enhances efficacy of CTLA-4 blockade in cancer immunotherapy. J Exp Med 2014;211:715–25. https://doi.org/10.1084/jem.20130590.

[8] Mandai M, Hamanishi J, Abiko K, Matsumura N, Baba T, Konishi I. Dual Faces of IFNγ in Cancer Progression: A Role of PD-L1 Induction in the Determination of Pro- and Antitumor Immunity. Clin Cancer Res 2016;22:2329–34. https://doi.org/10.1158/1078-0432.CCR-16-0224.

[9] Aspeslagh S, Postel-Vinay S, Rusakiewicz S, Soria J, Zitvogel L, A M. Rationale for anti-OX40 cancer immunotherapy. Eur J Cancer 2016;52:50–66. https://doi.org/10.1016/J.EJCA.2015.08.021.

[10] Szabo S, Sullivan B, Stemmann C, Satoskar A, Sleckman B, LH G. Distinct effects of T-bet in TH1 lineage commitment and IFN-gamma production in CD4 and CD8 T cells. Science 2002;295:338–42. https://doi.org/10.1126/SCIENCE.1065543.

[11] Burris H, Infante J, Ansell S, Nemunaitis J, Weiss G, VM V, et al. Safety and Activity of Varlilumab, a Novel and First-in-Class Agonist Anti-CD27 Antibody, in Patients With Advanced Solid Tumors. J Clin Oncol 2017;35:2028–36. https://doi.org/10.1200/JCO.2016.70.1508.

[12] Suntharalingam G, Perry MR, Ward S, Brett SJ, Castello-Cortes A, Brunner MD, et al. Cytokine Storm in a Phase 1 Trial of the Anti-CD28 Monoclonal Antibody TGN1412. N Engl J Med 2006;355:1018–28. https://doi.org/10.1056/nejmoa063842.

[13] Irenaeus S, Nielsen D, Ellmark P, Yachnin J, Deronic A, A N, et al. First-in-human study with intratumoral administration of a CD40 agonistic antibody, ADC-1013, in advanced solid malignancies. Int J Cancer 2019;145:1189–99. https://doi.org/10.1002/IJC.32141.

[14] Luke J, Barlesi F, Chung K, Tolcher A, Kelly K, A H, et al. Phase I study of ABBV-428, a mesothelin-CD40 bispecific, in patients with advanced solid tumors. J Immunother Cancer 2021;9. https://doi.org/10.1136/JITC-2020-002015.

[15] Cohen E, Pishvaian M, Shepard D, Wang D, Weiss J, ML J, et al. A phase Ib study of utomilumab (PF-05082566) in

combination with mogamulizumab in patients with advanced solid tumors. J Immunother Cancer 2019;7. https://doi.org/10.1186/S40425-019-0815-6.
[16] Zheng L, Judkins C, Hoare J, Klein R, Parkinson R, Wang H, et al. 812 Urelumab (anti-CD137 agonist) in combination with vaccine and nivolumab treatments is safe and associated with pathologic response as neoadjuvant and adjuvant therapy for resectable pancreatic cancer. J Immunother Cancer 2020;8:A862–A862. https://doi.org/10.1136/JITC-2020-SITC2020.0812.
[17] Segal N, Logan T, Hodi F, McDermott D, Melero I, O H, et al. Results from an Integrated Safety Analysis of Urelumab, an Agonist Anti-CD137 Monoclonal Antibody. Clin Cancer Res 2017;23:1929–36. https://doi.org/10.1158/1078-0432.CCR-16-1272.
[18] Heinhuis K, Carlino M, Joerger M, Di Nicola M, Meniawy T, S R, et al. Safety, Tolerability, and Potential Clinical Activity of a Glucocorticoid-Induced TNF Receptor-Related Protein Agonist Alone or in Combination With Nivolumab for Patients With Advanced Solid Tumors: A Phase 1/2a Dose-Escalation and Cohort-Expansion Clinic. JAMA Oncol 2020;6:100–7. https://doi.org/10.1001/JAMAONCOL.2019.3848.
[19] A Dose Escalation and Combination Immunotherapy Study to Evaluate BMS-986226 Alone or in Combination With Nivolumab or Ipilimumab in Patients With Advanced Solid Tumors - Full Text View - ClinicalTrials.gov n.d. https://clinicaltrials.gov/ct2/show/NCT03251924 (accessed September 8, 2021).
[20] Gutierrez M, Moreno V, Heinhuis K, Olszanski A, Spreafico A, M O, et al. OX40 Agonist BMS-986178 Alone or in Combination With Nivolumab and/or Ipilimumab in Patients With Advanced Solid Tumors. Clin Cancer Res 2021;27:460–72. https://doi.org/10.1158/1078-0432.CCR-20-1830.
